# Animal Models of *Trypanosoma cruzi* Congenital Transmission

**DOI:** 10.3390/pathogens11101172

**Published:** 2022-10-11

**Authors:** Eduardo E. Avalos-Borges, Lizette E. Rios, Matilde Jiménez-Coello, Antonio Ortega-Pacheco, Nisha J. Garg

**Affiliations:** 1 Departamento de Salud Animal y Medicina Preventiva, Facultad de Medicina Veterinaria y Zootecnia, Universidad Autónoma de Yucatan, Merida 97000, Yucatan, Mexico; 2 Department of Microbiology & Immunology, University of Texas Medical Branch, 301 University Boulevard, Galveston, TX 77555-1070, USA

**Keywords:** *Trypanosoma cruzi*, Chagas disease, congenital transmission, murine models, placenta, dogs

## Abstract

Chagas disease, initiated by the etiological agent *Trypanosoma cruzi*, is an endemic infection in the American continent. Although vectorial transmission of *T. cruzi* is recognized as the main mode of infection, other routes such as congenital and blood transfusion are also documented as important methods of transmission. *T. cruzi* maternal–fetal transmission has been recorded in humans and examined by some investigators in naturally and experimentally infected mammals. Dogs are recognized as the major reservoir host in maintaining the domestic transmission of *T. cruzi*; however, the importance of congenital transmission in preserving the infection cycle in dogs has not been studied in detail. In this article, we reviewed the current knowledge of congenital transmission of *T. cruzi* in humans and compared the placental architecture of humans and different animals with particular attention to rodents, dogs, and non-human primates that have been used as experimental models of *T. cruzi* infection, congenital transmission, and Chagas disease pathogenesis. The placentas of humans and animals have some similar and dissimilar characteristics that should inform the study design and interpretation of results when evaluating the efficacy of new anti-parasite drugs and therapies against congenital infection.

## 1. Introduction

The *Trypanosoma cruzi* (discovered by Chagas in 1909) (*Tc*) pathogen is the causative agent of Chagas disease (CD), also known as American Trypanosomiasis (AT) [1]. *T. cruzi* is a flagellate parasite of the phylum Euglenozoa, class Kinetoplastida, family Trypanosomatidae, and genus *Trypanosoma*, and it is known to infect humans and various species of wild and domestic mammals [1]. Trypanosomes are unicellular organisms with a single nucleus located in the center of the body. The infectious trypomastigote form of *T. cruzi* is typically spindle-shaped with an undulating membrane along its axis and a DNA-rich mitochondrial organelle called kinetoplast located in the anterior part of the parasite [2]. A series of microtubules originating near the kinetoplast make up the basal body that extends along the undulating membrane to the opposite end of the parasite. The flagellar tubules are surrounded by a membrane, and together they form the flagellar pocket [3]. Trypanosomes contain many of the same organelles as noted in their mammalian host, but also have organelles, e.g., glycosome, acidocalcisome, cytoplasmic vacuole, and reservosome, that are unique to these parasites [3,4].

Patients exposed to *T. cruzi* encounter a high parasitic load in circulation and various tissues for 1–2 months, after which it is significantly controlled by the adaptive immune response, and then intermittent low level of parasites are observed during the indeterminate phase of the disease [5]. Progression to clinically symptomatic chronic disease phase in ~30–40% of the infected patients is presented with the development of cardiomyopathy, though other morbidities including gastroenterological and neurological disorders are also noted in Chagas patients. Chagas disease can ultimately cause death due to heart failure [6].

*T. cruzi* populations survive in a wide ecological range and exhibit a highly diversified genetic profile. By analyzing a variety of molecular markers, *T. cruzi* clones have been categorized into seven discrete typing units (DTUs) referred as TcI-TcVI and Tcbat [7,8,9,10]. The parasite isolates belonging to these DTUs differ in geographical distribution and epidemiological association, as well as in virulence, pathogenicity, and tissue tropism in the host [11,12,13]. In Mexico and Central America, *T. cruzi* isolates of TcI DTU are found with the highest prevalence [14], though mixed infections with TcII, TcV, and TcVI are also noted in Mexico and Central America [15]. *T. cruzi* isolates of TcI and TcII DTUs with some cases of TcIV, TcV, and TcVI DTUs are noted in Brazil [16] and Argentina [11,17]. TcI-TcVI DTUs have been reported in large number of vectors species and mammals, Tcbat is transmitted from infected bats, and all DTUs can cause Chagas disease in humans and animals, though some evidence indicates an association between DTUs and clinical outcomes [18]. Readers interested in more details of geographic distribution of *T. cruzi* lineages in mammalian and vectorial hosts are referred to recent review articles [9,19].

The World Health Organization estimates that approximately 6–7 million people are affected by Chagas disease worldwide, and nearly 100 million people live in the endemic areas with high risk of exposure to *T. cruzi* infection in the Latin American countries [20,21]. The main form of transmission is by contact with feces of infected triatomine insects. Infection occurs when *T. cruzi*-infected triatomines feed on the host, contaminated feces from the insect is excreted, and parasites gain access to the blood stream of the host via skin puncture or mucous membranes [22]. Parasite transmission may also occur by oral ingestion of infected bugs [23], transfusion of infected blood [24], and congenital transmission from infected mothers [25]. After the vector control programs implemented in 1980–1990s were highly successful in controlling the acute transmission of *T. cruzi* from infected bugs in South America, the non-vectorial transmission pathways have emerged as major issues of public health concern [24]. In fact, congenital transmission has led to the globalization of Chagas disease in non-endemic countries [26,27,28,29]. It is projected that internationally over two million women of reproductive age are infected with *T. cruzi*, and 1–10% of fetuses carried by infected mothers are born with CD [30,31,32].

In this review, we summarized the current knowledge regarding *T. cruzi* congenital transmission in humans and animal models with an aim to point out the similarities and dissimilarities of placental and maternal factors between animals and humans. It is our hope that the current review will serve as an essential source of knowledge for understanding the pathophysiology of congenital transmission and permit the reader to make an informed decision when using experimental models of *T. cruzi* infection for studying the pathomechanisms of congenital transmission or examining the efficacy of new diagnostic tests or therapies to prevent congenital CD.

## 2. Diagnosis and Burden of Congenital Transmission of *T. cruzi* in Humans

Women, irrespective of being in the acute or chronic phases of CD, can congenitally transmit the parasite to the fetus and newborn [33]. Yet, there is significant evidence that a high rate of transmission occurs when pregnant women are acutely infected and/or develop reactivated acute infection due to immunosuppression [34]. Indeed, ~53% of women who became infected during pregnancy exhibited prenatal or perinatal transmission of *T. cruzi* to their fetuses and newborns [34,35,36,37]. Likewise, women infected with *T. cruzi* prior to pregnancy who became exposed to HIV or were treated with immunosuppressive drugs during gestation transmitted *T. cruzi* with high frequency to their newborns [38,39]. Besides, high parasite burden in umbilical cord of infants is associated with the severity of congenital CD [31,40]. In Mexico, studies indicate that an estimated seroprevalence of 2.21% (95% CI 1.46–2.96) would result in 50,675 births from *T. cruzi*-infected pregnant women and ~3193 cases of vertical transmission and infected neonates per year [41]. It is of note that infected mothers transmitted the same DTUs of *T. cruzi* to their newborns as were predominantly identified in the local population [17,42,43]. When pregnant women were exposed to mixed or multiclonal infections, predominance of different clones in the mother and their newborns has been noted [42,44,45]. Overall, the current literature allows us to surmise that (a) all DTUs can potentially be transmitted via congenital route, (b) natural selection of the transmitted parasite may occur during pregnancy, and (c) diagnostic screening of pregnant women and newborns and identification of parasite lineage would inform the timely treatment of newborns and restrict *T. cruzi* transmission.

The criteria for the occurrence of congenital transmission in humans are that the mother is *T. cruzi*-seropositive, parasites are detectable in the peripheral blood of the newborn, and anti-*T. cruzi* antibodies are made in the newborn after passive immunity acquired during lactation has disappeared (if vector and blood transfusion infection have also been ruled out) [37,46]. Microscopic observation of parasite in fresh blood smears offers the simplest approach to diagnosis of *T. cruzi* infection in newborns [47]. When there is a low number of blood parasites, concentration methods such as Strout or micro Strout test are useful [48,49]. Another form of *T. cruzi* detection is micro hematocrit, which may be more convenient for parasite detection because of its simplicity, low cost, and capacity to detect 40 parasites/mL with 97.4% sensitivity [50]. However, parasite identification with micro hematocrit requires ~30 min per sample evaluation by trained personnel, and even then, this method identifies only 40–60% of the congenital transmission in newborns [46,51]. Indirect parasite detection methods such as hemoculture or xenodiagnosis, although sensitive, have the disadvantage in that they require several weeks for positive *T. cruzi* identification [48,52].

In infants of nine months age or older, serological detection of anti-*T. cruzi* antibodies by indirect hemagglutination assay (IHA) [53], indirect immunofluorescence (IIF) assay [54,55], or enzyme-linked immunosorbent assay (ELISA) [52,53] can be employed. Serology is routinely applied for diagnosis of *T. cruzi* infection in clinical laboratories in Latin America [48,52,56]; however, difficulty in following up the newborns is a major limitation of this method in diagnosing and treating the infected infants in a timely manner.

The application of conventional polymerase chain reaction (PCR) for positive or negative diagnosis of congenital *T. cruzi* infection has increased in recent years [48,56,57]. When used at birth, PCR test provides higher sensitivity and specificity compared with other parasitological identification methods [37,58]. Indeed, accuracy of PCR test in diagnosis of congenital *T. cruzi* infection has been demonstrated in several studies [56,59,60,61]. A variation of PCR is the quantitative PCR (qPCR), which allows enumerating the parasite burden [62]. The qPCR assay based on *T. cruzi* satellite DNA and kinetoplast DNA can detect 0.85 and 0.43 parasite equivalents per mL blood, respectively, making it a highly sensitive approach [63]. The main drawback of this technique is the requirement for specific laboratory instrument and well-qualified employees who maintain rigorous quality control. As an alternative, loop-mediated isothermal amplification (LAMP) can be performed using a heat-block at a constant temperature of 60–65 °C and it does not require specialized PCR equipment [64,65]. LAMP has been successfully implemented to amplify *T. cruzi* DNA with a similar sensitivity as was noted with qPCR [64,66]. One caveat in implementation of molecular assays in the field is that trained personnel are needed to accurately identify infected newborns and avoid false-positives due to contamination of maternal parasite DNA. Readers are directed to a recent review discussing the efficacy and implementation of old and new diagnostic tests and an ideal algorithm for diagnosis of congenital transmission of *T. cruzi* in infants [25].

## 3. Characteristics and Classification of the Mammalian Placental Barrier

The placenta is a temporary organ formed during pregnancy. It primarily functions in anchoring the fetus to the uterine wall and mediating the immune tolerance to avoid immunological rejection of the fetus while also maintaining the anti-infectious capacity [67,68,69]. Placenta is also required for the transfer of nutrients such as amino acids, lipids, and glucose to the fetus, and the exchange of oxygen, carbon dioxide, and fetal waste excretion [70].

**Figure 1:**
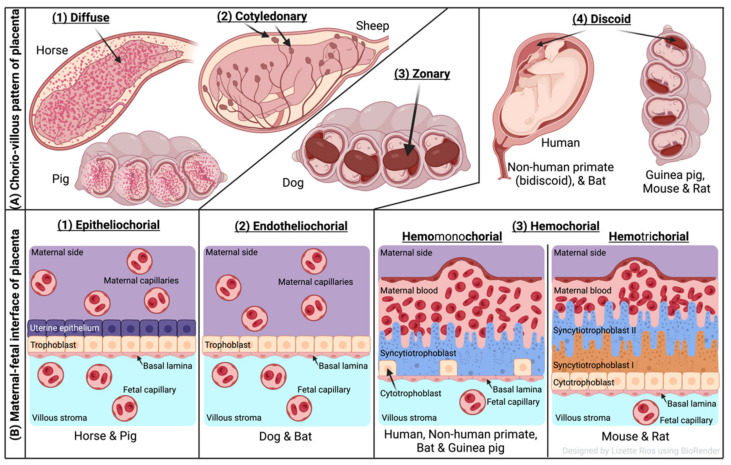
Comparative features of mammalian placenta. (**A**) Chorio-villous patterns of placenta. There are four types of placentas that are recognized: (1) diffuse: occurs over the entire surface of the uterine luminal epithelium with formation of folds/villi (seen in horses and pigs); (2) cotyledonary: characterized by many spot-like placental regions of the endometrium known as caruncles or cotyledons with smooth and poor vascularized intervening areas in the chorion (seen in ruminants); (3) zonary: placenta shows an intimate interdigitating contact zone that forms a belt or girdle around the chorionic sac (seen in carnivores such as dogs); (4) discoid/bidiscoid: characterized by a single (discoid) or double disc (bidiscoid), and mother–product interaction is confined to a roughly circular area (seen in humans, rodents, bats, and primates). (**B**) Maternal–fetal interface of placenta. This classification of placentas depends on the cell layers between the tissues of the mother and the fetus. (1) Epitheliochorial placenta (seen in horse, pig, and ruminants) is the least intimate because the interaction between the maternal blood and the fetal tissue is limited by a layer of uterine epithelial cells and a layer of trophoblast cells. (2) Endotheliochorial type (seen in dogs and cats) is the second more invasive barrier, where the uterine epithelium in degraded after implantation, leaving to the trophoblast adjacent only to the maternal endothelium. (3) Hemochorial barrier (hemomonochorial in primates and hemotrichorial in rodents) is the most intimate interface. The hemochorial epithelial and endothelial cells of the mother are degraded, leaving the trophoblast cells (syncytiotrophoblast and cytotrophoblast) in direct contact with the maternal blood.

The two forms of placenta classification are based on gross shape and histological structure (Figure 1). Gross morphology based placental classification describes whether maternal–fetal exchange occurs on all of the available surface of the chorionic sac or if it is restricted to specific zones [71,72]. Accordingly, four types of placentas categorized based on gross shape are shown in Figure 1A. These include (1) diffuse placenta, which appears over the complete surface of the uterine luminal epithelium with the formation of folds and villi, and it is seen in horses and pigs; (2) cotyledonary placenta, which consists of numerous spot-like placental regions of the endometrium known as caruncles or cotyledons with smooth and avascular intervening areas in the chorion, and it is seen in ruminants; (3) zonary placenta, which shows an intimate interdigitating contact zone that forms a strap or girdle around the chorionic sac, and it is seen in carnivores such as dogs, cats, bats, seals and bears; and (4) discoid/bidiscoid placenta, which may contain a single (discoid) or double (bidiscoid) disc, wherein maternal–placental interaction occurs in a roughly circular area, and it is seen in humans, rodents, rabbits, and primates [71,73].

Three main types of placental classification were proposed by Grosser based on histological structure (Figure 1B) [74]. This classification recognizes the histologic relationship of the chorion and uterine wall, and therefore, it is more apt and informative in describing the placental function [71,72]. The epitheliochorial type of placenta (seen in horses, pigs, and ruminants) is most superficial or least intimate because the maternal blood—fetal tissue interactions are limited by layers of uterine epithelial cells and fetal trophoblast cells only. In endotheliochorial placenta, withdrawal of maternal uterine epithelium and connective tissue after implantation informs the maternal endometrial contact with fetal trophoblasts. The endotheliochorial type of placenta is seen in four major clades of eutherian mammals, including carnivores, dogs, and cats. Lastly, the hemochorial placenta is considered most invasive, as in this case maternal hemochorial epithelial and endothelial cells are degraded, and thereby maternal blood is in open exchange with fetal trophoblast cells (syncytiotrophoblast and cytrotrophoblast). The hemomonochorial, hemodichorial, and hemotrichorial placentas consist of one, two, and three trophoblast layers, and are noted in primates, rabbits, and rodents, respectively [73,75].

## 4. Human Placental Barrier and *T. cruzi* Invasion

**Figure 2:**
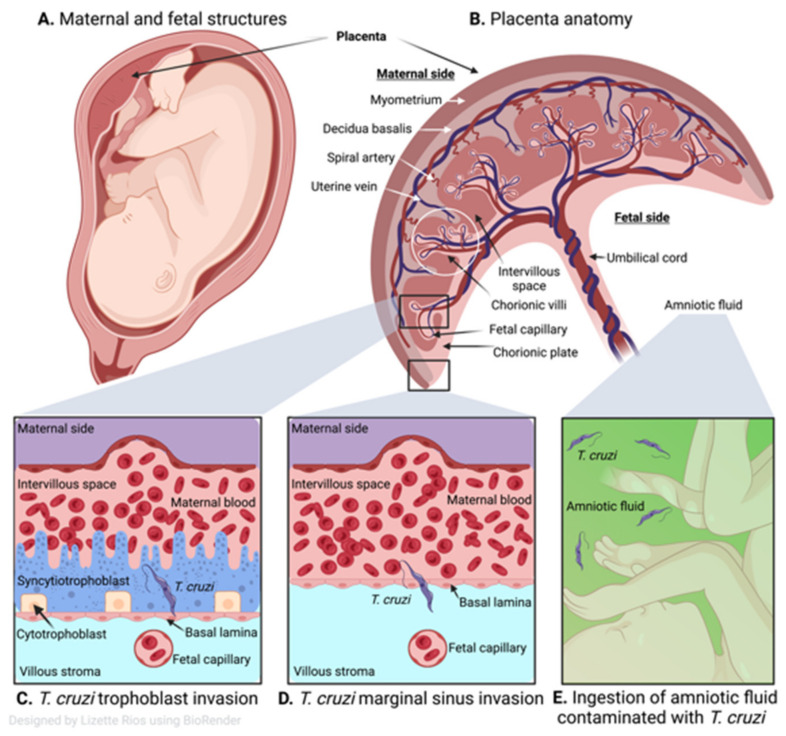
Human placenta and congenital transmission of *T. cruzi.* (**A**) Human maternal and fetal structures. (**B**) Human placental anatomy showing maternal side consisting of the myometrium, decidua basalis, spiral artery, uterine vein, intervillous space and the fetal side with chorionic villi, fetal capillary, chorionic plate, umbilical cord, and amniotic fluid. (**C**) *T. cruzi* infects different types of trophoblasts of the placental chorionic villous (cytotrophoblasts and syncytiotrophoblasts), then invades the capillaries of the umbilical cord and the fetus. (**D**) Marginal sinus of the chorionic plate is deprived of trophoblasts so the parasite can readily cross and once it reaches the chorion and exceeds the mesenchymal (villous stroma), it can gain access to the umbilical cord vessels and fetal capillaries. (**E**) Parasites that reach the amniotic fluid (green) after replicating in the placenta can contaminate the fetus orally.

The general anatomy of the human placenta is presented in Figure 2A,B. The characteristic maternal and fetal structures of a hemochorial discoid placentation are observed within the uterus (Figure 2A), where a direct contact occurs between the fetal trophoblastic cells and the maternal blood (Figure 2B). At a cellular level, the maternal–fetal interface of placenta in humans is established by decidua, trophoblastic, and chorioamniotic cells. Fetal trophoblasts differentiate into cytotrophoblast (CTB), syncytiotrophoblast (STB), and extravillous trophoblast that form the placental villi and interact with maternal blood present in the intervillous space [68]. The chorionic villi made up of syncytiotrophoblasts (first line of cells in contact with maternal blood), cytotrophoblasts (internal trophoblastic cells), basal lamina (basement membrane), the villous stroma or mesoderm (extracellular matrix), and fetal vascular system establish a direct interaction with maternal blood in the intervillous space [68].

Some studies indicate that congenital transmission occurs when *T. cruzi* dismantles the placental barrier developed by the trophoblast, basal laminae, and villous stroma, and then invade the capillaries of the umbilical cord to reach the fetus (Figure 2C) [76,77]. Histological analysis of human placentas showed diffused and severe villitis with *T. cruzi* nests, trophoblastic necrosis, and inflammatory infiltrate were increased when seropositive women gave birth to stillborn as compared with live births [78]. Thus, trophoblast invasion by *T. cruzi* is accepted as the most common route of parasite transmission to fetal tissues [79]. Others have proposed that congenital transmission occurs when *T. cruzi* crosses the placental barrier, especially at the marginal sinus of the chorionic plate (Figure 2D). Fernandez-Aguilar et al. [80] found a propensity of parasitic lesions in placental tissues of infected newborns at the marginal sinus that gradually decreased towards chorionic plate and distant membranes. Since the marginal sinus is deprived of trophoblasts, the parasite can readily reach chorion and mesenchymal (villous stroma) cells, and gain access to the umbilical cord vessels to infect the fetal tissues. It has also been proposed that parasites reach the amniotic fluid after replicating in the placenta and orally contaminating the fetuses (Figure 2E) [34,81].

How *T. cruzi* breaches the placental barrier and avoids the multifaceted immune defense mounted by the mother, placenta, and fetus to cause congenital infection remains understudied. Several parasite virulence factors including placental tropism, exovesicles, gene polymorphism, placental defense mechanisms, and the maternal and neonatal immune reaction are identified as potential factors in modulating the risk of congenital transmission, and these are discussed in recent reviews [82,83,84,85]. Briefly, chorionic villi in human placental explants lost their resistance to *T. cruzi* infection when STB layer was stripped off, thus implying the importance of first structure of the placental barrier in fetal protection [83,84,85]. Moreover, placenta is recognized as an active immunological organ and expresses proinflammatory and anti-inflammatory cytokines to provide protection against infectious agents, while avoiding fetus rejection [86,87]. Reprogramming of human placental genes expression of the innate immune pathways was noted in response to *T. cruzi* infection [88]. Nitric oxide produced by syncytiotrophoblasts is relevant for the placental protection against *T. cruzi* [89]. However, excessive production of reactive oxygen species, reactive nitrogen species, and inflammation-stimulating cytokines in the intervillous space may also injure the placental barrier [84,89] and allow parasite entry. In human placental chorionic villi explants, *T. cruzi* induced IL-6 and IL-10 cytokines that dysregulated the trophoblast turnover [90]. Moreover, parasite surface proteins, e.g., gp85 and gp83, which bind to glycoproteins of the extracellular matrix such as laminin, fibronectin, and heparan sulphate may support parasite attachment to the placenta [91]. Others have indicated that parasite can degrade collagens (type I and type IV) by secretion of proteases such as cruzipain to enter various cells [92]. Placenta from *T. cruzi*-infected women showed reorganization of extracellular matrix tissue and trophoblast cells, which was speculated to occur in conjunction with inflammatory immune activation in the host [91]. In summary, vertical transmission in most cases results from elevated maternal parasitemia that cannot be controlled by the immune response of the mother and placental defense (Table 1). Yet, several factors, including parasite (strain, burden, and virulence), placental integrity, and the quality and quantity of anti-parasite immunity play a complex role in determining the extent by which congenital transmission of *T. cruzi* would occur.

## 5. Rodent Models of Congenital Infection by *T. cruzi*

Rats and mice have discoid, hemotrichorial placentas that are closest to human placentas [107]. For this reason, and because of short gestation period with large litter size, and ease of handling due to small size, rodents offer the most cost-effective, biologically relevant experimental models to study the congenital transmission of *T. cruzi* (Table 1). Other advantages are the analogous expression of genes in mouse and human placenta [108], ability to use sophisticated imaging techniques to longitudinally monitor pregnancy in rodents [109], and availability of a wide variety of genetically modified mice and immunological reagents to study mouse response to pregnancy in the presence and absence of infection [108]. Yet, mice and humans exhibit differences in placental endocrine functions, gestation length, and fetal developmental stages [110]. Further, because of the shallowness of trophoblasts, particularly at the blood vessels of the decidua that remain lined with endothelium [109,111], mouse models are not optimal for studying the trophoblast invasion and vascular remodeling that play a critical role in fetal growth restriction and pre-eclampsia in pregnant women [107].

Wistar rats (*Rattus norvegicus*) have been widely employed for studying the *T. cruzi* maternal–fetal infection. In acutely infected, pregnant female Wistar rats, placentas associated with fetuses showed modest infiltration of immune cells and no parasites were detected in the vascular stroma and amniotic fluid. Yet, 9.1% of the offspring developed high parasitemia at 30–40 days after birth that was detectable by blood microscopic examination, hemoculture, and xenodiagnosis [93]. The congenitally infected offspring also developed acute myocarditis and myositis characterized by abundant inflammatory infiltrate and muscle weakness that was associated with amastigote nests in some cases [93]. In another study, offspring of acutely infected Wistar dams did not show blood parasitemic phase until 60 days after birth, but a progressive increase in serum levels of anti-*T. cruzi* IgM was noted in 24% of the pups between 15 and 60 days after birth, while maternal anti-*T. cruzi* IgGs decreased progressively [94]. When Wistar dams infected with *T. cruzi* (Y strain) were mated in chronic phase, blood analysis for parasites by light microscopy, blood culture, and xenodiagnosis revealed no indication of patent or sub-patent parasitemia in mothers and offspring, while anti-*T. cruzi* antibodies were present in 100% of pregnant dams and 44.6% of their offspring by IIF and ELISA-based serology tests [95]. Histological examination of maternal tissue sections revealed parasite persistence in skeletal muscle, myocardium, and placental villi; muscle weakness; and mild-to-moderate inflammatory infiltrate in the heart, uterus, umbilical cord, and mammary glands [95]. *T. cruzi* trypomastigotes (flagellated, infective form) were detected in cardiac tissue of some of the fetuses which also presented an intense inflammatory infiltrate in their placenta [96]. In another study, congenitally infected Wistar rats born to acutely infected mother dams were mated, and second-generation offspring were examined by various parasitological tests. The second-generation offspring exhibited *T. cruzi* negativity by direct blood tests and blood culture, 18.2% positivity by xenodiagnosis, 31.8–34% positivity by serology tests for anti-*T. cruzi* antibodies, and 45.4–54.5% positivity by PCR analysis of heart and skeletal muscle [112]. Together, these findings indicate that the presence of infective form of *T. cruzi* and anti-parasite IgM antibodies in the blood and *T. cruzi* DNA in blood or tissues can be used to confirm the congenital infection in the progeny. Congenital dissemination of *T. cruzi* in second-generation offspring presents a startling challenge to the transmission control efforts.

In NMRI female mice injected with *T. cruzi* (Y strain) and mated during acute infection phase, a decline in the number of fetuses, morphological and structural abnormalities of fetuses, and low birth weight and growth retardation of the newborns were noted, as compared with the normal, healthy pregnant mice [97,98]. Lesions characterized by protuberances on dorsal side of the body and on the footpad were found in fetuses of acutely infected female mice. Histology showed infiltration of macrophages, monocytes, and lymphocytes in cardiac fibers in 10% of the newborns. Presence of *T. cruzi* antigens in placenta and skeletal muscle of fetuses exhibiting morphological alterations was also noted [97,98]. In BALB/c mice acutely infected with *T. cruzi* (TcI: X10 strain, TcII: Y strain, and TcVI: Tulahuen strain), parasite dose and route of parasite delivery led to varying consequences for fetuses [99,100]. While females acutely infected with low dose of TcVI Tulahuen strain were able to develop immune resistance and prevent congenital transmission [99], females injected with high dose of either of the three parasite strains within a few days after mating or close to delivery exhibited intrauterine growth retardation and pups’ mortality [100]. Gestation during chronic infection with any of the three strains also resulted in intrauterine growth retardation, and pups’ mortality was increased if chronically infected females were re-inoculated with the Tulahuen strain [100]. Likewise, BALB/c mice exhibited different pregnancy outcomes depending on the stage of parasite exposure with Tehuantepec strain isolated from triatomines in Mexico. BALB/c females acutely infected with Tehuantepec strain (100 trypomastigotes) exhibited severe infertility and early fetal loss [101]. Remaining fetuses of the infected females exhibited reduced birth weight, extensive tissue inflammatory infiltrate, and necrosis in several organs, and all fetuses died within two days after birth. Massive foci of parasite associated with inflammatory infiltrate and ischemic necrosis, fibrin deposits, and vascular thrombosis were detected in decidua and other placental tissues of the fetuses born to acutely infected female mice [101]. In contrast, BALB/c female mice infected with *T. cruzi* Tehuantepec strain and mated during chronic phase exhibited similar fertility rate/placental weights as were noted in non-infected/pregnant mice, and similar parasitemia as noted in non-pregnant/infected controls. Parasites were not detected in blood of the fetuses of chronically infected/pregnant mice, yet the fetal weight was significantly decreased [102]. de Araujo et al. [103] reported that even if chagasic female mice (C3H/He) exposed to low-dose parasite did not transmit infection to newborns, offspring of the infected mothers were more susceptible to subsequent challenge infection than the offspring of the non-infected mothers, thus suggesting that maternal immune components can modulate the offspring’s immune response.

Only a few investigators have used Swiss outbred white mice for studying the pathogenesis of congenital CD. Swiss female mice infected with Morc-I strain of *T. cruzi* (DTU Tcbat isolated from an infected bat) exhibited parasite foci in uterine muscles and decidual and endothelial placental cells, intrauterine developmental delays, 10% fetal mortality, and transplacental transmission in 30% of the fetuses [104]. Andrade [105] challenged female Swiss mice with four different isolates of *T. cruzi* and observed incidences of placental parasitism were highest (98%) in females infected with TcIII Colombian strain, while 13–18% placental parasitism was observed in females infected with TcI (Y or Peruvian strain) or TcII (Honorina strain) DTUs. As above, Swiss females chronically infected with Tulahuen strain exhibited low-to-no transplacental passage of anti-*T. cruzi* maternal antibodies and congenital transmission [106].

Altogether, studies in rats and mice demonstrate that acute *T. cruzi* exposure resulting in parasite infiltration and placental necrosis can cause infertility, fetal loss, and impaired reproduction. It is proposed that invasion and replication capacity of the parasite also determines the extent of its effect on reproduction and congenital transmission.

## 6. Congenital Infection in Guinea Pigs and Chiropters

Guinea pigs (*Cavia porcellus*) exhibit long gestation periods and deliver precocial pups, and many events of fetal development in guinea pigs are similar to those noted during human gestation. This contrasts with rats and mice, which give birth to altricial pups after short gestation period and undergo many of the maturation processes during postnatal development. Thus, guinea pigs offer a better model than mice and rats for studying the fetal development [113]. This is especially encouraging as the guinea pig genome has recently been sequenced and annotated, and biotechnology companies have taken interest in developing a large number of antibodies to study immunological responses of guinea pigs in detail. Besides, guinea pigs’ physiology and endocrinology of gestation is very well described, including the normal expected fetal growth [113], which facilitates the understanding of the effects of infectious agents.

Sherlock and Muniz [114] demonstrated that successive transmission of *T. cruzi* could occur in three generations of the guinea pigs without exposure to infected triatomines. More recently, we infected guinea pigs with low dose of a virulent H4 strain of *T. cruzi* (TcI DTU) before or during gestation, and noted that irrespective of the parasite exposure stage, infected dams transmitted parasite to 100% of the offspring, determined by qPCR [62]. Fetuses of mothers infected during pregnancy showed higher levels of myocardial and skeletal tissue lesions and necrosis than was observed in fetuses of females infected before pregnancy. Placental infiltration of inflammatory infiltrate and parasitic foci were negligible when examined by histology; however, qPCR analysis clearly identified placental parasites in all infected guinea pigs. Compromised fetal development, evidenced by decreased birth weight and reduced body size, was noted in all infected guinea pigs [62].

Bats, in general, receive negative public attention, yet some investigators have begun to examine the natural history of *T. cruzi* infection in bats and their importance as a reservoir host in zoonotic transmission of the parasite. In pregnant bats (*Molosus molosus*) captured from a natural habitat in Venezuela, congenital *T. cruzi* transmission to bats’ offspring was documented by hemoculture and PCR methods. PCR assay detected *T. cruzi* DNA in cardiac tissue of 80% of the captured pregnant bats and 100% of their fetuses, thus demonstrating that bats are highly susceptible to *T. cruzi* [115]. The captured bats were infected with *T. cruzi* TcI DTU that is also predominantly detected in human populations in Venezuela, Colombia, Brazilian Amazonia, and Mexico, thus suggesting that bats might contribute to endemicity of *T. cruzi* in Latin American countries. Congenital transmission of Chagas disease in bats can add additional risk to parasite circulation in domestic cycle as their natural habitats are destroyed with new developments and constructions, increasing bats’ displacement to urbanized areas [116].

## 7. Congenital Transmission in Other Mammals

There are significant differences in the structure and physiology of the placenta between different species of larger mammals. Each of these can provide new insights for the congenital transmission of Chagas disease as a study model for humans, and to complement the epidemiology of this disease in endemic areas where these species are found. For example, primates, due to their great resemblance to the human placenta, offer a promising model for studying the pathogenesis of human congenital transmission, but their acquisition and maintenance cost make them an unviable study model [117]. Other, more accessible larger mammals are domestic dogs, which have longer gestations and larger litter sizes. However, the placentation (zonary, endotheliochorial) is different from that of humans, which limits its use as an experimental model [118].

An outdoor-enclosed colony of the rhesus macaques (*Macaca mulatta*) at a biomedical research organization in Texas, USA was sampled to identify gestational difficulties consistent with congenital CD. An overall 3.9% of the rhesus macaques at this site were naturally infected with *T. cruzi*, evidenced by the presence of anti-*T. cruzi* antibodies, but no differences in gestational outcomes were found when comparing seropositive (*n* = 62) and seronegative (*n* = 1021) females over a period of four years [119]. However, the study was not specifically designed to examine the pathogenesis of congenital Chagas disease in rhesus macaques.

Vertical transmission of *T. cruzi* in dogs (*Canis familiaris*) has been reported in a few studies. Campos reported as early as in year 1928 the disseminated presence of parasite in different organs of newborn pups of acutely and chronically infected female dogs. However, not all the pups of the same litter were infected [120]. In a pair of animals (female and male) that were infected four months before mating with the TcI Ninoa strain of *T. cruzi*, parasitemia was not detected at the onset of the clinical signs (lymph adenomegaly and fever) by direct observation of fresh blood smears at 10–21 days post inoculation. Yet, the newborn puppies exhibited muscle weakness, low growth rate, and chronic diarrhea. Necropsy and histopathological studies revealed digestive and cardiac disorders and tissue inflammatory infiltrate in all puppies. Anti-parasite antibodies were detected in parents and offspring by an ELISA and IIF assay, and parents presented significantly higher titers of anti-*T. cruzi* antibodies than the puppies examined at 47 days after birth [121]. Altogether, this study suggested that despite the inability to detect parasite and maternal antibodies in the pups born to infected dogs, pups developed *T. cruzi*-induced sickness. More recently, we found that pregnant dogs naturally infected with *T. cruzi* can show a transmission rate of 0.59, where 20% of the litter can be infected (unpublished data).

## 8. Conclusions

The studies of congenital CD in the different animal models seem to be contradictory and inconsistent even in the same species. However, these differences may be influenced by the lineage of the parasite, inoculated dose, the stage of the infection during gestation, as well as the immune status of the mother and the characteristics of the placenta. In species that represent important reservoirs such as dogs and bats, congenital transmission has not been convincingly demonstrated. More studies are required in dogs due to their great abundance and capacity to become important reservoirs that coexist closely with humans and other domestic or peridomestic animal species.

## Figures and Tables

**Table 1:** Maternal fetal transmission of *T. cruzi* in experimental models.

Species/Model	*T. cruzi* Strains	*T. cruzi* Dose	Route	Disease	Transmission Rate	Observations	References
Guinea pigs	H4	100 blood trypomastigotes	IP	Acute Chronic	100%	Cell degeneration and necrosis in myocardial and skeletal tissues of fetuses, compromised fetal development	[62]
Human placenta explant	Tulahuen	8 × 10^4^ metacyclic trypomastigotes	NA	NA	NA	Chorionic villi denuded of syncytiotrophoblasts were more susceptible to infection.	[83,84,85]
Human placenta explant	NA	1 × 10^4^ metacyclic trypomastigotes	NA	NA	NA	Reprograming of human placental genes expression of the innate immune pathways by *T. cruzi* infection.	[88]
Human placenta explant	Tulahuen or Lucky	1 × 10^5^ or 1 × 10^6^ metacyclic trypomastigotes	NA	NA	NA	Nitric oxide produced by syncytiotrophoblasts are relevant for the placental protection against *T. cruzi.*	[89]
Human placenta explant	NA	1 × 10^5^ metacyclic trypomastigotes	NA	NA	NA	*T. cruzi* induced IL-6 and IL-10 dysregulated the trophoblast turnover.	[90]
Human placenta	NA	NA	NA	Asymptomatic	2/3 *	Placenta from *T. cruzi*-infected women showed reorganization of extracellular matrix tissue and trophoblastic cells and inflammatory immune response.	[91]
Wistar rats	Pr or YBM	1 × 10^4^ metacyclic trypomastigotes	ID	Acute	9.1% 47.8%	Acute myocarditis, amastigote nests in fetal hearts.	[93]
Wistar rats	Planalto	1.5 × 10^4^ metacyclic trypomastigotes	IP	Acute	24%	Parasite antigens detected in heart and skeletal muscle of 15% of pups.	[94]
Wistar rats	Y	1 × 10^5^ blood trypomastigotes	ID	Chronic	44.6%	Virulence and inoculum size determines extent of vertical transmission of *T. cruzi.*	[95]
Wistar rats	Planalto	1 × 10^5^ blood trypomastigotes	IP	Chronic	33%	Amastigote nests in fetal cardiac tissues, inflammatory infiltrate in placentas.	[96]
NMRI mice	Y	22 × 10^3^ metacyclic trypomastigotes	IP	Acute	15%	Structural abnormalities in fetuses, loss of weight and growth retardation in pups, myocarditis in 10% of pups.	[97]
NMRI mice	Y	22 × 10^3^ metacyclic trypomastigotes	IP	Acute	18%	Inflammatory infiltrate and amastigote nests in fetal hearts.	[98]
BALB/c mice	Tulahuen	150 blood trypomastigotes	IP	Acute	0%	Female resistance to *T. cruzi* infection prevented congenital transmission.	[99]
BALB/c mice	X10, Y or Tulahuen	1 × 10^6^ blood trypomastigotes	SC	Acute	4%	Intra-uterine growth retardation of fetuses.	[100]
BALB/c mice	Tehuantepec	100 blood trypomastigotes	IP SC	Acute	0%	Placental invasion by parasites and injury, fetal growth retardation and death in the absence of congenital infection, 28% of embryos resorbed in early gestation.	[101]
BALB/c mice	Tehuantepec	100 blood trypomastigotes	IP SC	Chronic	NA	Parasites were not detected in fetuses of chronically infected mice, yet fetal weight was significantly decreased.	[102]
C3H/He mice	Y	500 blood trypomastigotes	IP	Acute	1.18%	Maternal immune products transferred by placental and suckling routes modulate fetal immune response.	[103]
Swiss mice	Morc-1	1 × 10^5^ blood trypomastigotes	IP	Acute	30%	Amastigote nests in uterine muscle, striated muscle, and placental cells; intrauterine growth retardation and mortality in 10% of fetuses.	[104]
Swiss mice	Y, Peruvian (P), Honorina (H), Colombian (C)	Y: 144–157, P: 463, H: 72–500, C: 473 trypomastigotes	SC	Acute Chronic	0% 0%	The incidence of placental parasitism-Y: 17%, H: 13.2%, C: 98%, Peruvian: 18.4%. Parasite strains play a role in congenital *T. cruzi* infection.	[105]
Swiss mice	Tulahuen	25–30 blood trypomastigotes	NA	Chronic	6.1%	Anti-*T. cruzi* maternal antibodies and parasite was rarely noted in newborns, myocytic lesions found in fetal tissues.	[106]

Maternal route of parasite inoculation is indicated as ID (intradermal), IP (intraperitoneal), SC (subcutaneous). * *T. cruzi* was detected by PCR in two out of three neonates delivered by asymptomatic mothers. NA: not available.

## Data Availability

All data are included or appropriately referenced in the article.
